# TRPM8 deficiency attenuates liver fibrosis through S100A9-HNF4α signaling

**DOI:** 10.1186/s13578-022-00789-4

**Published:** 2022-05-07

**Authors:** Qiang Liu, Xiaohua Lei, Zhenyu Cao, Ju Zhang, Likun Yan, Jie Fu, Qing Tong, Wei Qin, Yaoli Shao, Chun Liu, Zhiqiang Liu, Zicheng Wang, Yuan Chu, Ge Xu, Siyuan Liu, Xueyi Wen, Hirofumi Yamamoto, Masaki Mori, Xin. M. Liang, Xundi Xu

**Affiliations:** 1 Hunan Provincial Key Laboratory of Hepatobiliary Disease Research, Division of Hepato-Biliary-Pancreatic Surgery, Department of Surgery, The Second Xiangya Hospital, Central South University, 139 Renminzhong Road, Changsha, 410011 People’s Republic of China; 2 Hunan Cancer Hospital, The Affiliated Cancer Hospital of Xiangya School of Medicine, Central South University, Changsha, 410013 People’s Republic of China; 3 Department of Surgery and Clinical Oncology, Graduate School of Medicine, Osaka University, Suita City, Osaka 565-0871 Japan; 4 Department of Surgery and Science, Graduate School of Medical Sciences, Kyushu University, Fukuoka, 812-8582 Japan; 5 Wellman Center for Photomedicine, Division of Hematology and Oncology, Division of Endocrinology, Massachusetts General Hospital, VA Boston Healthcare System, Beth Israel Deaconess Medical Center, Harvard Medical School, Boston, MA 02115 USA; 6 Present Address: Department of General Surgery, Health Science Center, South China Hospital, Shenzhen University, No.1, Fuxin Road, Pinghu Street, Longgang District, Shenzhen, 518116 People’s Republic of China

**Keywords:** TRPM8, Liver fibrosis, ECM, Inflammation, S100A9, HNF4α

## Abstract

**Background:**

Liver fibrosis represent a major global health care burden. Data emerging from recent advances suggest TRPM8, a member of the transient receptor potential (TRP) family of ion channels, plays an essential role in various chronic inflammatory diseases. However, its role in liver fibrosis remains unknown. Herein, we assessed the potential effect of TRPM8 in liver fibrosis.

**Methods:**

The effect of TRPM8 was evaluated using specimens obtained from classic murine models of liver fibrosis, namely wild-type (WT) and TRPM8^−/−^ (KO) fibrotic mice after carbon tetrachloride (CCl_4_) or bile duct ligation (BDL) treatment. The role of TRPM8 was systematically evaluated using specimens obtained from the aforementioned animal models after various in vivo and in vitro experiments.

**Results:**

Clinicopathological analysis showed that TRPM8 expression was upregulated in tissue samples from cirrhosis patients and fibrotic mice. TRPM8 deficiency not only attenuated inflammation and fibrosis progression in mice but also helped to alleviate symptoms of cholangiopathies. Moreover, reduction in S100A9 and increase in HNF4α expressions were observed in liver of CCl_4_- and BDL- treated TRPM8^−/−^ mice. A strong regulatory linkage between S100A9 and HNF4α was also noticed in L02 cells that underwent siRNA-mediated S100A9 knockdown and S100A9 overexpressing plasmid transfection. Lastly, the alleviative effect of a selective TRPM8 antagonist was confirmed in vivo.

**Conclusions:**

These findings suggest TRPM8 deficiency may exert protective effects against inflammation, cholangiopathies, and fibrosis through S100A9-HNF4α signaling. M8-B might be a promising therapeutic candidate for liver fibrosis.

**Graphical Abstract:**

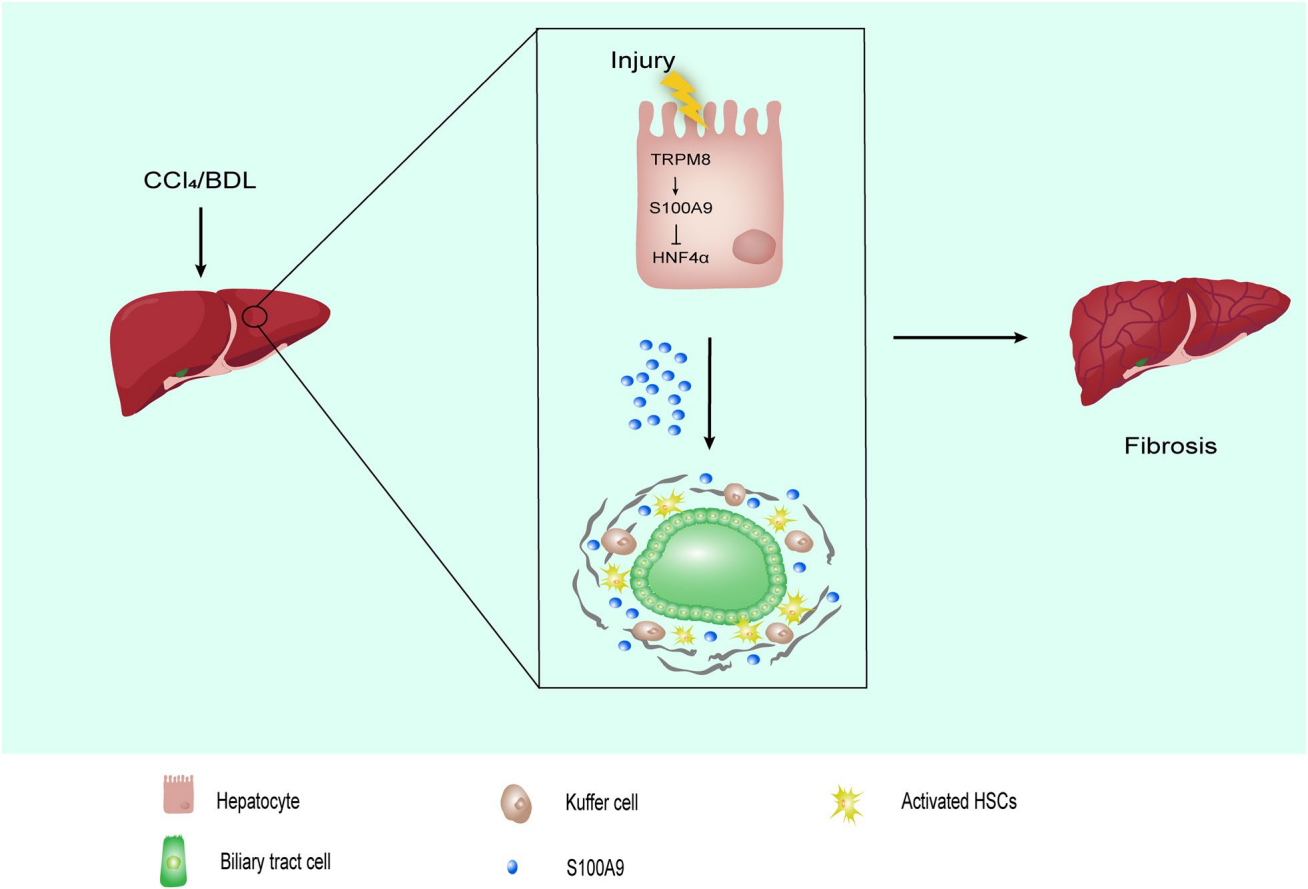

**Supplementary Information:**

The online version contains supplementary material available at 10.1186/s13578-022-00789-4.

## Introduction

Liver fibrosis, a major health care burden worldwide, represents the ubiquitous response of the liver to almost all chronic injuries predominantly arising from sustained viral infection, excessive alcohol consumption, metabolic etiologies, and autoimmune liver diseases [1, 2]. It is commonly characterized by the activation of hepatic stellate cells (HSCs) and excess accumulation of extracellular matrix (ECM) [3]. Despite global efforts in developing therapeutic interventions to treat this devastating disease, our understanding of the complex mechanism of liver fibrosis remains limited and no acceptable treatment strategies currently exist. There is an urgent clinical need to better comprehend the underlying mechanisms and identify effective antifibrotic agents aimed at impeding or reversing liver fibrogenesis [4, 5].

Fibrogenesis is usually triggered by either chronic inflammation and oxidative stress or repeated death of hepatocytes or biliary tract cells, promoting fibroblasts proliferation and ECM production at the injured site [6]. Previous studies have suggested that continuous inflammation from resident and infiltrating immune cells plays an important role in the liver fibrotic process [7]. In particular, liver macrophages, such as Kupffer cells and monocyte-derived infiltrating macrophages, have been proven to be major players during fibrogenesis by promoting the activation and survival of HSCs [8, 9]. Furthermore, during the recovery phase of chronic liver injury, the regression of liver fibrosis is often accompanied by a distinct restorative macrophage subpopulation [10]. Taken together, accumulating evidence identifies that macrophage-mediated inflammation is a vital factor that influences the progression of liver fibrosis.

Cholangiocytes, the epithelial cells lining the intrahepatic and extrahepatic bile ducts, are highly dynamic pivotal cells residing in a complex anatomic niche where they participate in hepatic canalicular bile modification, bile production, and homeostasis [11, 12]. Cholangiocytes show increased proliferation in response to endogenous or exogenous signals/stimuli and are actively involved in inflammatory and reparative processes within the liver. Sustained stimulatory insults to these cells may induce the initiation and progression of cholangiopathies [13–15]. To date, the roles of liver inflammation and fibrosis in cholangiopathies remain obscure.

Transient receptor potential (TRP) cationic channels are known to be activated by various physical and chemical stimuli to promote signal transduction [16], among which TRPM8 is especially interesting because it can be activated by environmental stimuli [17, 18], play a critical role in numerous cancers [19] and inflammation-related diseases [20, 21]. However, its role in the pathogenesis of liver fibrosis remains unclear.

Here, we report the functional significance of TRPM8 in liver fibrosis. The obtained data show an aberrantly upregulated TRPM8 expression in both fibrotic human clinical and mice specimens. A strong correlation between TRPM8 deficiency and alleviated liver fibrosis progression was also observed in CCl_4_- and BDL-treated mice. In addition, we noticed TRPM8 ablation could attenuate various symptoms of cholangiopathy, including bile canalicular abnormalities, dysregulation of bile transporters, and ductular reaction. Further, transcriptomic analyses of mRNAs using fibrotic mice liver specimens, coupled with both in vitro and in vivo assays, were conducted to understand the underlying molecular mechanism of TRPM8 in liver fibrosis. The results from this study suggest TRPM8 inhibition may lead to suppression of liver fibrogenesis through S100A9-HNF4α signaling axis, which raises the possibility that TRPM8 might be served as a potential therapeutic target for anti-fibrosis therapy development.

## Materials and methods

### Human serum and liver samples

Serum samples were obtained from 15 patients with clinically diagnosed cirrhosis and 10 healthy donors. Liver tissues were obtained from 10 patients with hepatic hemangioma (non-fibrotic samples) and 40 patients with cirrhosis who underwent liver transplantation at the Second Xiangya Hospital of Central South University. All study related experimental protocols were under the ethical guidelines of the 1975 Declaration of Helsinki Principles and were approved by the Ethics Committee of the Second Xiangya Hospital of Central South University. Written informed consent was obtained from all human subjects. Healthy donors with no previously known liver abnormality and who had not received any medication in the two weeks prior to sample collection participated in this study.

### Reagents

CCl_4_ (C112040) and olive oil (O108686) were purchased from Aladdin (Shanghai, China). The hematoxylin and eosin (H&E) staining kit (G1120), Sirius Red staining kit (G1471), and Masson’s trichrome staining kit (G1340) were purchased from Solarbio (Beijing, China). TRIzol reagent was purchased from Invitrogen (Thermo Fisher SCIENTIFIC, MA, USA). A Universal Two-Step Test Kit (PV-9000) was purchased from ZsBio (Beijing, China). The following antibodies were used: anti-TRPM8 (GTX54866, GeneTex, California, USA), anti-α-smooth muscle actin (α-SMA) (BM0002, Boster, Beijing, China), anti-collagen type I (COL1A1) (BA0325, Boster, Beijing, China), anti-cytokeratin 19 (CK19) (GB11197, Servicebio, Wuhan, China), anti-β-actin (60008-1-AP, Proteintech, Rosemont, USA), anti-F4/80 (28463-1-AP, Proteintech, Rosemont, USA), anti-S100A9 (14226-1-AP, Proteintech, Rosemont, USA), and anti-HNF4α (ab41898, Abcam, Cambridge, UK).

### Animals and animal experiments

WT and TRPM8^−/−^ (systemic knockout) C57BL/6J mice were obtained from Jackson Laboratory (ME, USA). All mice received humane care throughout experiments according to the guidelines of Central South University. All animal experiments were approved by the University Committee on Use and Care of Animals.

CCl_4_ is a hepatotoxic substance, and the CCl_4_-induced liver fibrosis model is one of the most common toxic liver fibrosis models. Six-week-old male WT and TRPM8^−/−^ mice were given intraperitoneal injections of CCl_4_ to mimic toxic-induced fibrosis (1.0 mL/kg body weight, 1:4 dissolved in olive oil) or vehicle (olive oil) twice a week for eight weeks (n = 5 per group). Two days after the last CCl_4_ injection, mice were sacrificed and specimens collected.

Persistent cholestasis can damage the liver, and the BDL-induced liver fibrosis model is an ideal model to study cholestatic liver fibrosis. Six-week-old male WT and TRPM8^−/−^ mice underwent ligation of the common bile duct (n = 5 per group) to mimic cholestatic liver fibrosis. Fourteen days after the BDL procedure, mice were sacrificed and specimens collected.

For therapeutic studies, CCl_4_-injured C57BL/6J mice received DMSO, 10 mg/kg M8-B hydrochloride, or 10 mg/kg WS-12 intraperitoneal injection [22] every other day from the 7th to the 8th week (n = 5 per group), while BDL-injured mice received either DMSO, 10 mg/kg M8-B hydrochloride, or 10 mg/kg WS-12 intraperitoneal injection every other day after one week of BDL treatment (n = 5 per group).

### Histology analysis

Formalin-fixed, paraffin-embedded 4 μm thick liver sections were stained with either H&E for cell morphology characterization or Sirius Red and Masson’s trichrome for quantitative assessment of liver fibrosis severity. Axio Scope A1 light microscopy (Carl Zeiss, Germany) was used to acquire digital images from random fields, and the most representative views of the sections are presented. Cell morphology and the severity of liver fibrosis were evaluated by experienced pathologists while blinded to the treatment groups.

### Transmission electron microscopy

Liver tissue obtained from CCl_4_-treated mice was thin-sectioned first followed by 2.5% glutaraldehyde fixation in phosphate-buffered saline by perfusion via the inferior vena cava. Transmission electron microscopy was performed as described previously [23].

### Immunohistochemistry (IHC)

The expressions of TRPM8, α-SMA, COL1A1, F4/80, CK19, S100A9, and HNF4α in the liver were evaluated using immunohistochemical staining. Briefly, the tissue sections were deparaffinized, hydrated, and incubated in 3% hydrogen peroxide, to block endogenous peroxidase. Antigen retrieval was performed by heating in 10 mM sodium citrate buffer (pH 6.0) for 20 min, and then incubation with primary antibodies against TRPM8 (1:200 dilution), α-SMA (1:500 dilution), COL1A1 (1:500 dilution), F4/80 (1:1000 dilution), CK19 (1:1000 dilution), S100A9 (1:400 dilution) or HNF4α (1:500 dilution) antibodies at 4 °C overnight. A universal two-step test kit was used to treat the samples after they had been incubated with appropriate primary antibodies. Finally, sections were counterstained with hematoxylin for 10 min, dehydrated and sealed with neutral balsam before a light microscope equipped (Olympus, Hamburg, Germany) with a digital camera was used to photograph the regions of interest. Three images at randomly selected locations were acquired for each tissue section.

### Serum biochemistry

Serum levels of alanine aminotransferase (ALT) and aspartate aminotransferase (AST) were measured using automatic biochemical analyzers (Abbott, Chicago, USA). Human serum S100A9 was measured using a S100A9 ELISA kit (CSB-E11834h, CUSABIO, Wuhan, China) following the manufacturer’s recommended procedure.

### Cell experiments

L02 cells were purchased from the Cell Bank of Xiangya Medical School, Central South University. L02 cells were cultured for 24 h before being exposed to TRPM8 siRNA, S100A9 siRNA (RiboBio, Guangzhou, China), or S100A9 plasmid (GeneChem, Shanghai, China). Lipofectamine™ 3000 transfection reagent was used to treat L02 cells following the manufacturer’s instructions. The transfection medium was replaced with complete culture medium after 6 h. Cells were collected for further investigations after 48 h.

Primary mouse hepatocytes were prepared from male WT and TRPM8^−/−^ mice following the well-established collagenase perfusion method [24]. After purifying the primary mouse hepatocytes, cells were collected for further investigations.

### qRT-PCR and RNA-seq analysis

Total RNA was extracted from frozen liver tissue using TRIzol reagent following the manufacturer’s instructions. Relative mRNA expression levels were calculated using the relative quantification method (2^−ΔΔCT^). All used PCR primer sequences can be found in Additional file 6: Table S1. A melting curve for each amplicon was determined to verify its specificity. Genes were normalized to β-actin as an internal control. Liver RNA samples were submitted for RNA-seq analysis at Annoroad Gene Technology Co., Ltd.

### Western blot analysis

Proteins in lysates were resolved by SDS–PAGE electrophoresis and transferred to PVDF membrane (Millipore Corporation, MA, USA). The blotted membrane was blocked and immunoblotted with TRPM8 (1:500 dilutions), α-SMA (1:500 dilutions), COL1A1 (1:500 dilutions), S100A9 (1:500 dilutions), and HNF4α (1:1000 dilutions) primary antibodies at 4 ℃ overnight. β-actin (1:5000 dilution) was used as a control. After washing with TBST, the membranes were incubated with secondary antibodies (1:5000 dilution) for 1 h at room temperature, followed by detection with enhanced chemiluminescence system. Image J was used to quantify the grey values for each target protein band.

### Statistical analysis

Data were expressed as mean ± SD and analyzed using GraphPad Prism 7. Statistical differences between two groups were analyzed by the unpaired t-test with a two-tailed distribution. Differences between multiple groups of data were analyzed by one-way ANOVA with Bonferroni correction. A *P*-value of less than 0.05 was considered to be statistically significant.

## Results

### TRPM8 expression is elevated in liver fibrosis

**Fig. 1:**
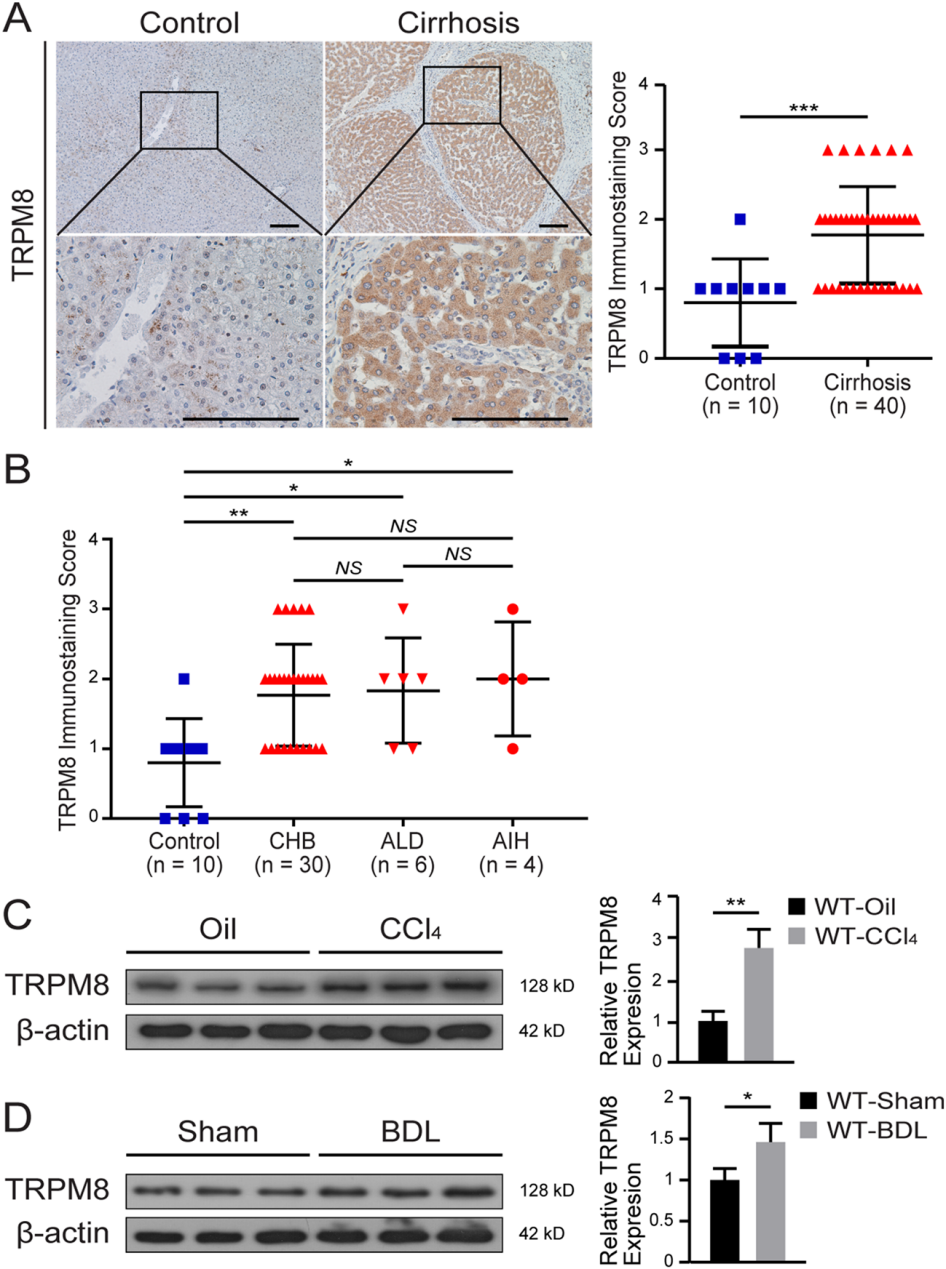
TRPM8 is highly expressed in the livers of cirrhotic patients and fibrotic mice. **A** Representative images of immunohistochemical staining of TRPM8 in healthy control and cirrhotic human liver tissues, and its statistical summary. Scale bars, 100 μm. **B** Cirrhotic patients were divided into three different categories according to their etiologies: chronic hepatitis B (CHB), alcohol-related liver disease (ALD), and autoimmune hepatitis (AIH). **C**, **D** Immunoblotting analysis of hepatic TRPM8 expression in CCl_4_- and BDL-induced liver fibrosis model (n = 3 per group). The results are expressed as the mean ± SD. *P < 0.05, **P < 0.01, ***P < 0.001

Immunohistochemical staining indicated TRPM8 expression in human cirrhotic liver tissue was significantly upregulated compared to healthy donors, and the increased TRPM8 expression in the liver was localized mainly to hepatocytes but not the α-SMA-expressing HSCs (Fig. 1A). The cohort of 40 patients included hepatitis B-related cirrhosis, alcoholic cirrhosis and autoimmune hepatitis-related cirrhosis. The demographics and clinical characteristics of human samples are presented in Additional file 6: Table S2. Immunohistochemical analysis also revealed an enhanced hepatic TRPM8 expression in cirrhotic tissues compared to normal human liver tissues, regardless of etiology (Fig. 1B). Western blot analysis further illustrated an elevated TRPM8 expression in both CCl_4_- and BDL-induced liver fibrotic mice (Fig. 1C, D). Taken together, these data suggest that TRPM8 may be involved in hepatic fibrogenesis.

### TRPM8 deficiency attenuates liver fibrosis in mice

**Fig. 2:**
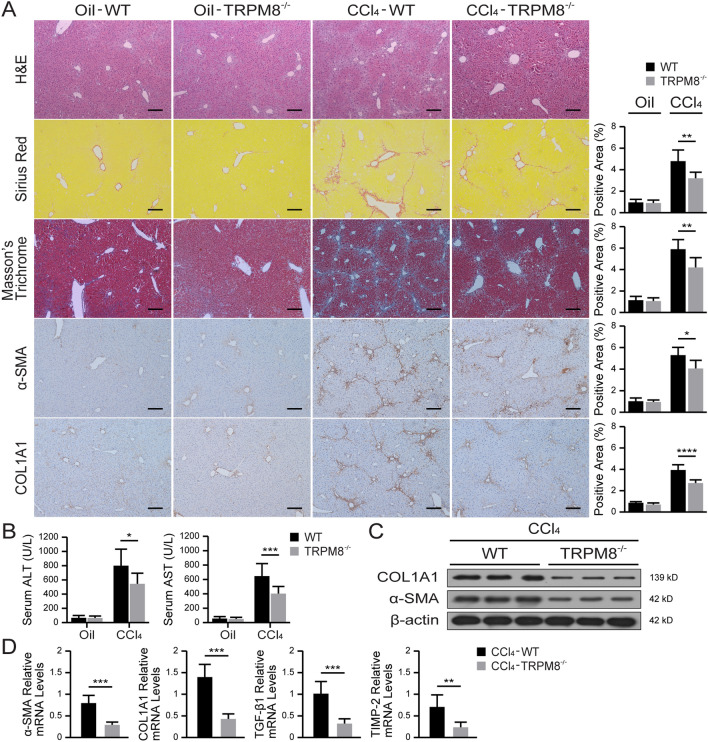
Liver fibrosis is attenuated in TRPM8^−/−^ mice after CCl_4_ treatment. **A** Representative histology of H&E, Sirius Red, Masson’s trichrome, and IHC staining for α-SMA and COL1A1 in the liver of WT and TRPM8^−/−^ mice induced by CCl_4_ (n = 5 per group). Positive staining areas were quantified by by Image J software. Scale bars, 100 μm. **B** Liver function was assessed by measuring the serum levels of ALT and AST in mice (n = 5 per group). **C** Immunoblotting analyses of α-SMA and COL1A1 expression in the mouse liver (n = 3 per group). **D** Hepatic mRNA levels of fibrogenic genes were measured by qRT-PCR (n = 5 per group). The results are expressed as the mean ± SD. *P < 0.05, **P < 0.01, ***P < 0.001, ****P < 0.0001

As illustrated in Fig. 2A, H&E, Sirius Red, and Masson’s trichrome stainings of the liver tissue all showed CCl_4_-induced TRPM8^−/−^ mice had significantly attenuated liver injury and fibrosis compared with WT mice. This finding was also supported by the significantly reduced α-SMA and COL1A1 expression in TRPM8 deficient mice. Moreover, liver injury, indicated by serum ALT and AST, was significantly reduced in TRPM8^−/−^ mice (Fig. 2B). Western blot analysis further showed suppressions of α-SMA and COL1A1 in the CCl_4_-induced TRPM8^−/−^ group compared to the WT (Fig. 2C). Additionally, CCl_4_-treated TRPM8^−/−^ mice had a greater reduction in hepatic mRNA expression of several profibrotic genes, such as α-SMA, COL1A1, TGF-β1, and TIMP-2 (Fig. 2D).

**Fig. 3:**
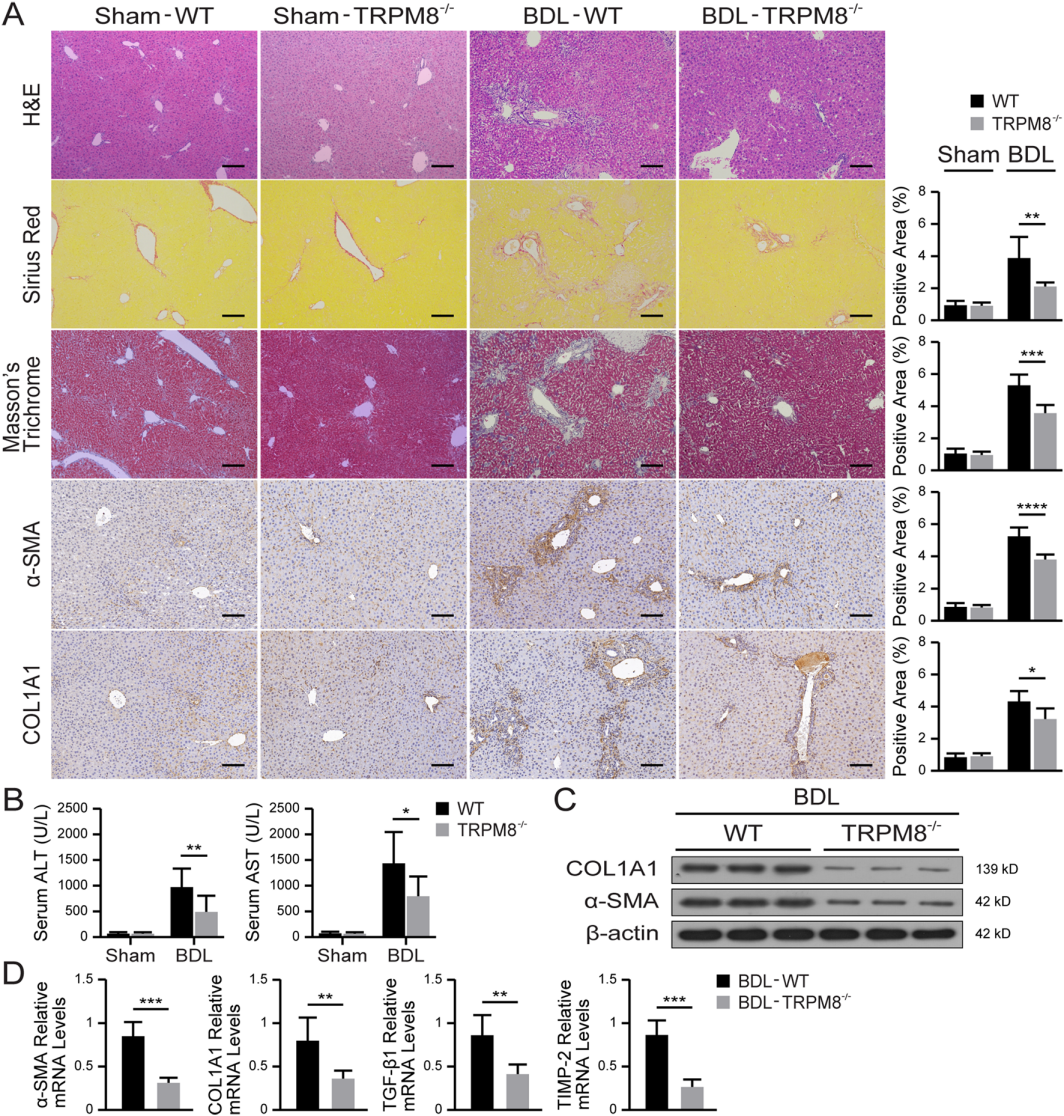
TRPM8 deficiency alleviates BDL-induced liver fibrosis in mice. **A** Representative images of H&E, Sirius Red, Masson’s trichrome, and IHC staining for α-SMA and COL1A1 in the liver of WT and TRPM8^−/−^ mice operated with BDL (n = 5 per group). Positive staining areas were quantified by Image J software. Scale bars, 100 μm. **B** Liver function was assessed by measuring the serum levels of ALT and AST in mice (n = 5 per group). **C** Immunoblotting analyses of α-SMA and COL1A1 expression in the mouse liver (n = 3 per group). **D** Hepatic mRNA levels of fibrogenic genes were measured by qRT-PCR (n = 5 per group). The results are expressed as the mean ± SD. *P < 0.05, **P < 0.01, ***P < 0.001, ****P < 0.0001

Similar histopathological staining of BDL-injured mice liver tissues also provided direct evidence that knocking out TRPM8 significantly alleviated fibrogenesis. The results of IHC (Fig. 3A) and Western blot (Fig. 3C) show greatly reduced α-SMA and COL1A1 expressions in TRPM8^−/−^ mice compared with the WT. In addition, remarkable reductions in serum ALT and AST levels were noticed in BDL-injured TRPM8^−/−^ mice (Fig. 3B), as were all the mRNA expressions of fibrogenic genes (Fig. 3D). These results indicate that TRPM8 deficiency observably improves liver fibrosis.

### TRPM8 deficiency ameliorates bile canalicular abnormalities, dysregulation of bile transporters, ductular reaction and liver inflammation in fibrotic liver

**Fig. 4:**
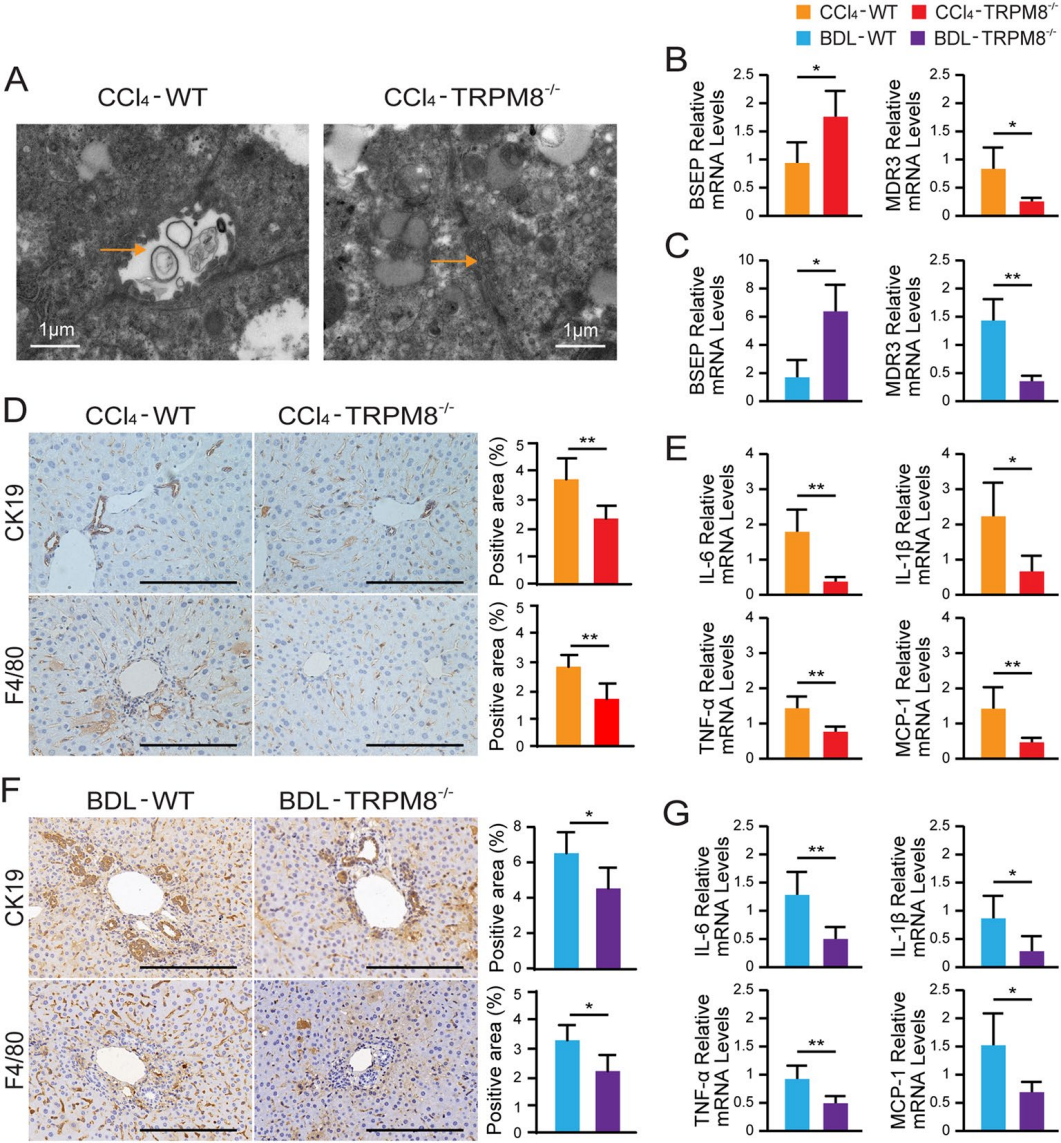
TRPM8 deficiency attenuates bile canalicular abnormalities, dysregulation of bile transporters, ductular reaction and liver inflammation in fibrotic liver. **A** Electron microscopy images show bile canaliculi (arrows) in liver sections obtained from CCl_4_-treated WT and TRPM8^−/−^ mice. **B**, **C** Hepatic mRNAs of bile acid transport genes were measured by qRT-PCR in liver specimens obtained from CCl_4_- or BDL-treated WT and TRPM8^−/−^ mice (n = 5 per group). **D**, **F** Histology of CK19 and F4/80 IHC staining in liver sections obtained from CCl_4_- or BDL-treated WT and TRPM8^−/−^ mice. Positive staining areas were quantified by Image J software. Scale bars, 100 μm. **E**, **G** Hepatic mRNAs of IL-6, IL-1β, TNFα, and MCP-1 were measured by qRT-PCR in liver specimens obtained from CCl_4_- or BDL-treated WT and TRPM8^−/−^ mice (n = 5 per group). The results are expressed as mean ± SD. *P < 0.05, **P < 0.01

Electron microscopy revealed not only irregularly dilated and tortuous bile canaliculi but also the lack of microvilli in CCl_4_-treated WT (Fig. 4A). Dysregulation of hepatic bile acid transporters, such as significantly lowered bile salt export pump (BSEP) mRNA and elevated MDR3 mRNA expression, was observed in both CCl_4_- and BDL-injured WT mice, as illustrated in Fig. 4B C, respectively. Furthermore, the levels of CK19 expression in liver tissues of both CCl_4_- and BDL-injured WT mice were significantly elevated compared to that of the injured TRPM8^−/−^ mice (Fig. 4D, F). Moreover, as ductular reaction is often associated with inflammatory cell infiltration in the portal region, the murine macrophage-specific F4/80 marker was used to study the inflammatory response of the liver. As expected, markedly more F4/80 positive cells were found in liver samples of WT fibrotic mice than that of the TRPM8^−/−^ groups (Fig. 4D, F). Consistently, significant reductions in transcription levels of inflammatory genes and chemokines (such as IL-6, IL-1β, TNFα, and MCP-1) were detected in liver tissues of both CCl_4_- and BDL-injured TRPM8^−/−^ mice (Fig. 4E, G).

### TRPM8 deficiency alleviates liver fibrosis by regulating S100A9-HNF4α signaling

**Fig. 5:**
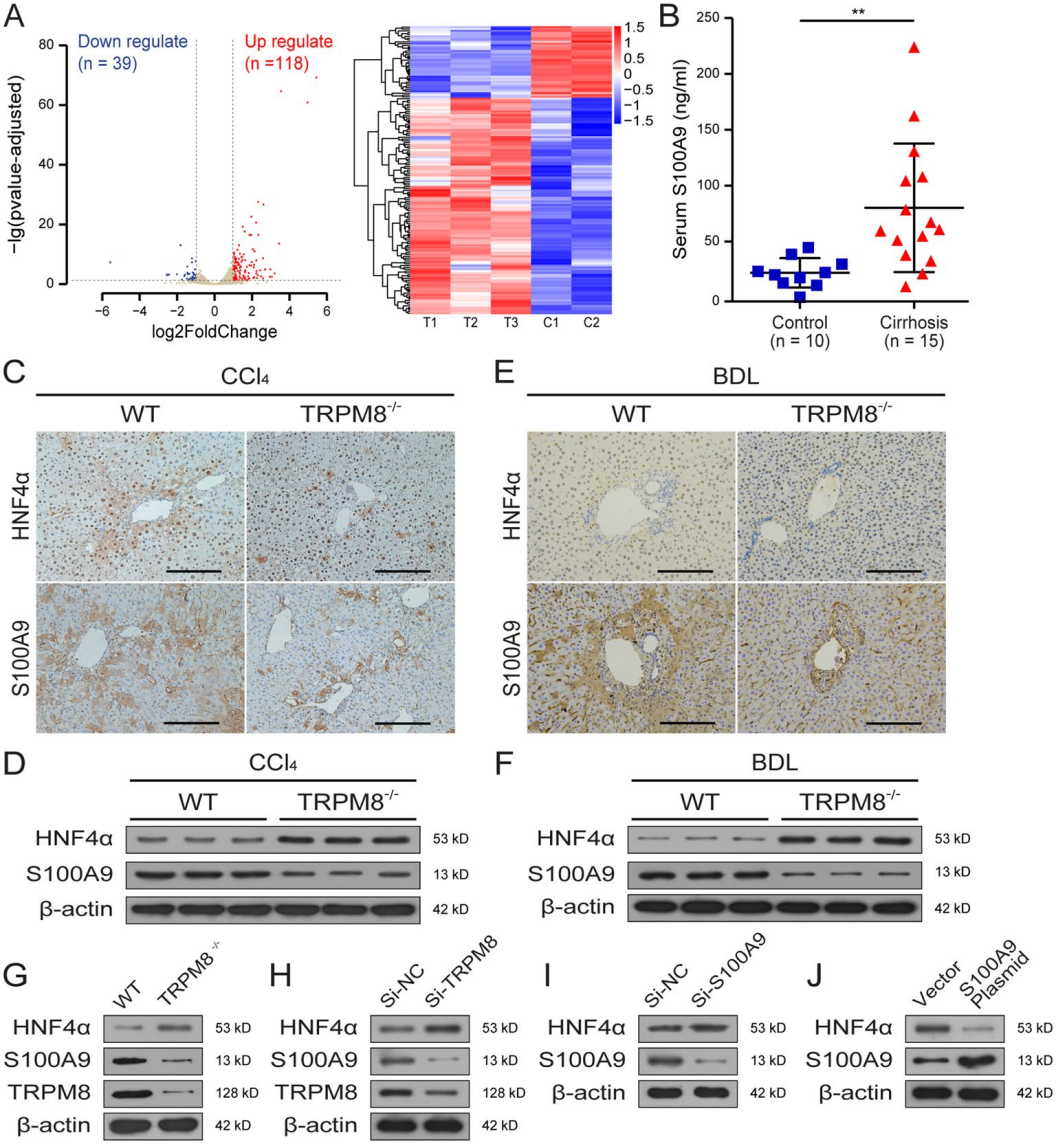
TRPM8 regulates the expression of S100A9 and HNF4α. **A** Volcano plot and heatmap presentation of significantly up- and downregulated genes in liver specimens obtained from CCl_4_-treated TRPM8^−/−^ mice compared to WT (n = 3 per group). **B** Serum S100A9 expressions in liver cirrhosis patients and healthy donors. **C**, **E** Histology of S100A9 and HNF4α IHC staining in liver sections obtained from CCl_4_- or BDL-treated WT and TRPM8^−/−^ mice (n = 5 per group). Scale bars, 50 μm. **D**, **F** Expressions of S100A9 and HNF4α were detected by immunoblotting using liver specimens obtained from CCl_4_- or BDL-treated WT and TRPM8^−/−^ mice (n = 3 per group). **G** Immunoblotting assays of TRPM8, S100A9, and HNF4α in primary hepatocytes from WT and TRPM8^−/−^ mice. **H** Immunoblotting assays of TRPM8, S100A9, and HNF4α in L02 cells transfected with TRPM8 siRNA after 48 h. **I**, **J** Immunoblotting assays of S100A9 and HNF4α in L02 cells transfected with S100A9 siRNA or plasmid after 48 h. The results are expressed as mean ± SD. **P < 0.01

After filtering the genes with a greater than two-fold change and a *P*-value of less than 0.05, transcriptomic analysis of mRNAs in CCl_4_-injured WT and TRPM8^−/−^ mouse liver revealed 118 upregulated and 39 downregulated genes. By Kyoto Encyclopedia of Genes and Genomes (KEGG) pathway enrichment analysis, these genes were mainly categorized into metabolism, hepatocellular carcinoma, chemical carcinogenesis, and inflammatory mediator regulation of TRP channels (Additional file 1: Fig. S1). Among them, genes are closely related to inflammation and fibrosis, such as S100A9, Col6a3 and CTGF, were found to be significantly reduced in TRPM8^−/−^ mice (Fig. 5A). Importantly, as the serum concentration of S100A9 is often elevated in patients with liver cirrhosis compared to healthy donors (Fig. 5B), we then have investigated the relationship between S100A9, an inflammation-related factor, and HNF4α, a well-known master regulator of liver-specific gene expression in fibrogenesis. The results of IHC and Western blot assays suggest TRPM8 ablation would lead to the downregulation of S100A9 and upregulation of HNF4α in CCl_4_-injured mice (Fig. 5C, D). A similar trend was also observed in liver specimens obtained from BDL-induced fibrotic mice (Fig. 5E, F). Furthermore, identical modulatory effects of TRPM8 on S100A9 and HNF4α expression were confirmed in primary hepatocytes isolated from both WT and TRPM8^−/−^ mice (Fig. 5G), as well as L02 cells (Fig. 5H). The negative regulatory relationship between S100A9 and HNF4α expression was subsequently demonstrated in L02 cells that underwent either siRNA-mediated S100A9 knockdown (Fig. 5I) or S100A9 overexpressing plasmid transfection (Fig. 5J). Taken together, these results imply TRPM8 deficiency may attenuate liver fibrosis by downregulating the expression of the pro-inflammatory factor S100A9 while promoting the expression of HNF4α.

### Inhibition of TRPM8 ameliorates murine liver fibrosis

**Fig. 6:**
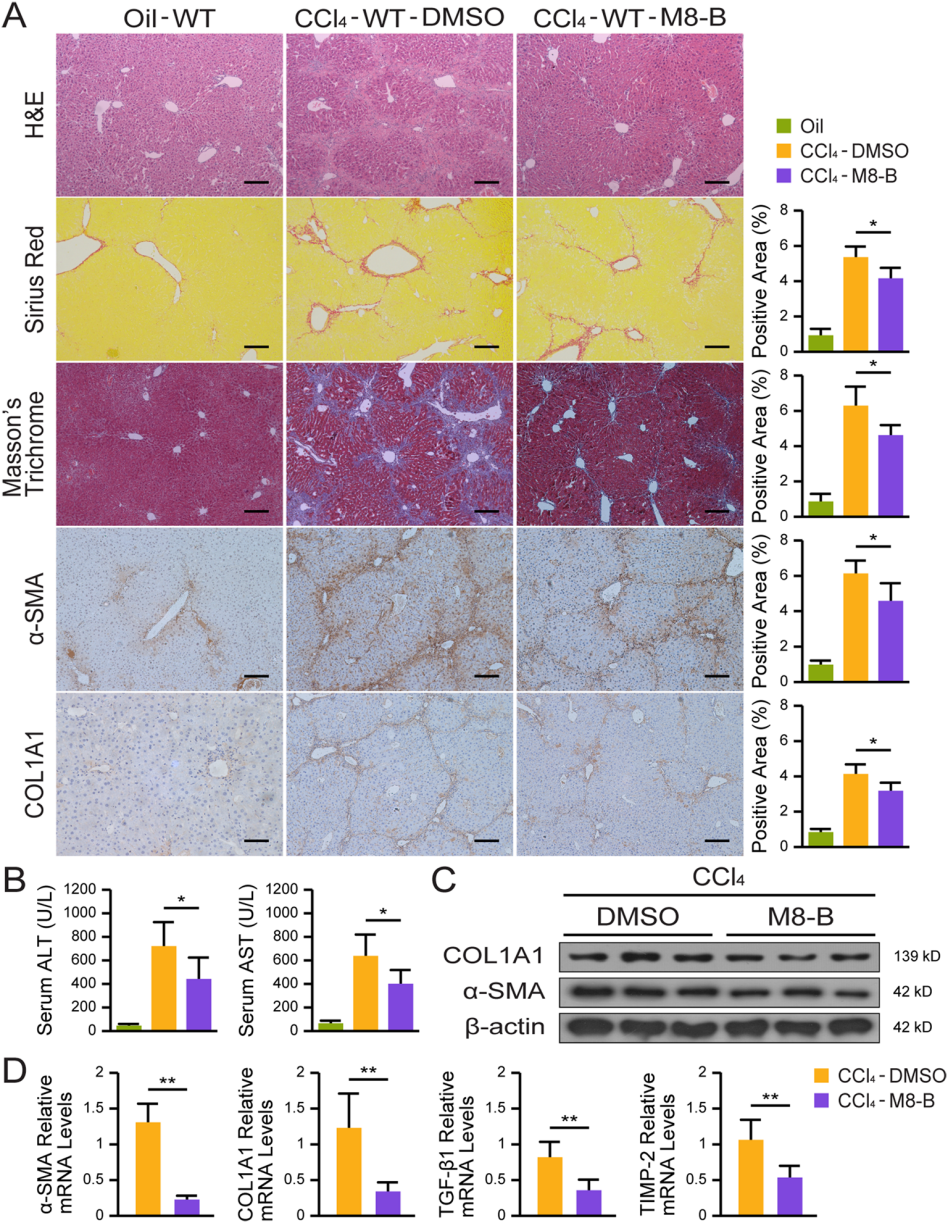
TRPM8 inhibitor mitigates liver fibrosis in CCl_4_-treated mice. **A** H&E, Sirius Red, Masson’s trichrome, and IHC staining for α-SMA and COL1A1 in liver sections of CCl_4_-treated mice (n = 5 per group). Image J was used to quantify positively stained areas. Scale bars, 100 μm. **B** Serum levels of ALT and AST were measured in mice (n = 5 per group). **C** Expressions of α-SMA and COL1A1 were detected by immunoblotting (n = 3 per group). **D** Hepatic mRNAs of fibrogenic genes were measured by qRT-PCR assays in mice treated with M8-B or DMSO after CCl_4_ induction (n = 5 per group). The results are expressed as mean ± SD. *P < 0.05, **P < 0.01

TRPM8 deficiency seemed to significantly attenuate the progression of liver fibrosis in fibrotic mice; thus, we next investigated the potential therapeutic effect of TRPM8 specific inhibitor (M8-B hydrochloride) and agonist (WS-12) on liver fibrosis. H&E, Sirius Red, and Masson’s trichrome staining assays verified M8-B treatment was able to delay the development of fibrosis induced by CCl_4_. Consistently, IHC staining also showed M8-B significantly reduced α-SMA and COL1A1 expression in the liver of fibrotic mice (Fig. 6A and Additional file 2: Fig. S2). Biochemical tests of the serum samples revealed M8-B markedly reduced both ALT and AST levels, indicating an improved liver functional state (Fig. 6B). Similarly, M8-B treatment led to a noticeable suppression in α-SMA and COL1A1 expression according to Western blot assay (Fig. 6C), as well as the mRNA levels of the pro-fibrogenic markers, α-SMA, COL1A1, TGF-β1, and TIMP-2 according to qRT-PCR detection (Fig. 6D). The identical treatment was given to BDL-induced liver fibrotic mice, yielding comparable data (Additional file 3: Fig. S3 and Additional file 4: Fig. S4). Furthermore, we validated the effect of TRPM8 on S100A9 and HNF4α in TRPM8 inhibitor-treated mice (Additional file 5: Fig. S5), and the results are consistent with our previous findings in TRPM8 knockout mice. In summary, these results strongly suggest inhibiting TRPM8 may be a promising therapeutic approach for treating liver fibrosis.

## Discussion

The present study showed that TRPM8 is upregulated in both human liver fibrosis tissues and specimens collected from two murine fibrosis models, namely the CCl_4_-induced and BDL-treated mice. Notably, the obtained data suggest genetic inactivation of TRPM8 is able to intervene and effectively attenuate the progression of liver fibrosis in mice. Furthermore, the treatment of M8-B, a specific inhibitor of TRPM8, demonstrated anti-fibrotic potential in CCl_4_- and BDL-induced murine models. These findings suggest that TRPM8 could be a novel therapeutic target for liver fibrosis.

Extensive studies have elucidated the key role of inflammation in the pathogenesis of liver fibrosis. Activation of proinflammatory cytokines and chemokines, including IL-6, IL-1β, TNFα, and MCP-1, can activate HSCs, promote ECM secretion and deposition, and then exacerbate liver fibrosis [7]. In this study, we found that TRPM8 inhibition could reduce the F4/80 positive cell population and inhibit the transcript levels of IL-6, IL-1β, TNFα, and MCP-1 in murine models of liver fibrosis. These findings suggest TRPM8 ablation may alleviate liver fibrosis through inhibiting the inflammatory response.

In addition to its role in fibrosis, inflammation has also been central in discussions related to cholangiopathies, partly owing to the current prevailing view that, although the activating injury event is unknown in many cases, features that characterize activated cholangiocytes include not only increased proliferation but, more importantly, elevated levels of pro-fibrotic and pro-inflammatory secretions as well [13, 15]. The results show that knockout of TRPM8 was effective in not only restoring irregularly dilated and tortuous bile canaliculi and dysregulated hepatic bile acid transporters such as BSEP and MDR3 but also attenuating ductular reaction, mainly in terms of a reduced number of activated cholangiocytes. In general, despite the lack of more in-depth investigation, these data lay the basic groundwork for hypothesizing that hepatic fibrosis alleviation might be achievable through an alternative route, that is TRPM8 blockage may lead to less pro-inflammatory cytokines secretion, which suppresses the activation of cholangiocytes and, thus, improves liver fibrosis.

Furthermore, RNA sequencing and morphological examination were conducted to reveal the molecular mechanism of TRPM8 in the pathogenesis of liver fibrosis. Molecules related to inflammation and cholangiopathies were screened and verified using both tissue samples and in vitro assays. Dysregulation of S100A9 and HNF4α was identified in genetic intervened murine fibrotic models. In vitro assays also elucidated targeting TRPM8 could give rise to S100A9-HNF4α signaling.

S100A9, a member of the S100 protein family, is released from either inflammatory cells or damaged cells and binds to toll-like receptor 4 or the receptor for advanced glycation end products, thereby promoting inflammatory response [25, 26]. A growing body of evidence suggests a strong association of S100A9 with various inflammatory and fibrotic diseases [27–29]. Our results have also shown S100A9 expression was significantly elevated in serum samples from cirrhosis patients compared to healthy donors, as well as a significant reduction in liver samples from CCl_4_- or BDL-treated TRPM8^−/−^ mice. Therefore, it is hypothesized that TRPM8 mediates the inflammatory response by regulating the expression of S100A9.

HNF4α, the central transcriptional regulator of hepatocyte gene expression, differentiation, and function maintenance, has been shown to have a central regulatory role in not only the development of biliary cancer [30, 31] but also the formation of microvilli, maintenance of normal liver function, and alleviation of liver fibrosis [32, 33]. In accordance with these findings, our data show that inhibition of TRPM8 upregulated the expression of HNF4α in both CCl_4_- and BDL-treated models, suggesting its involvement in the process of liver fibrosis. At the same time, in vitro analysis also confirmed that S100A9 negatively regulates the expression of HNF4α in L02 cells. These data suggest that TRPM8 deficiency upregulates the expression of HNF4α via the downregulation of S100A9 expression. Certainly, additional studies will be needed to illustrate the detailed mechanism.

In conclusion, our results suggest that TRPM8 inhibition may alleviate the liver fibrosis caused by inflammation and cholangiopathies through S100A9-HNF4α signaling. TRPM8 may be a potential therapeutic target for liver fibrosis.

## Supplementary Information

**Additional file 1:** **Fig. S1.** KEGG pathway and GO enrichment analysis.

**Additional file 2:** **Fig. S2.** The effects of TRPM8 agonists on CCl_4_-induced liver fibrosis in mice. H&E, Sirius Red, Masson’s trichrome, and IHC staining for α-SMA and COL1A1 in liver sections of CCl_4_-treated mice (n = 5 per group). Image J was used to quantify positively stained areas. Scale bars, 100 μm. Results are expressed as mean ± SD.

**Additional file 3:** **Fig. S3.** TRPM8 inhibitor reduces fibrogenesis in BDL-treated mice. **A** H&E, Sirius Red, Masson’s trichrome, and IHC staining for α-SMA and COL1A1 in liver sections of BDL-treated mice (n = 5 per group). Image J was used to quantify positively stained areas. Scale bars, 100 μm. **B** Serum levels of ALT and AST were measured in mice (n = 5 per group). **C** Expressions of α-SMA and COL1A1 were detected by immunoblotting (n = 3 per group). **D** Hepatic mRNAs of fibrogenic genes were measured by qRT-PCR assays in mice treated with M8-B or DMSO after BDL induction (n = 5 per group). The results are expressed as mean ± SD. *P < 0.05, **P < 0.01.

**Additional file 4:** **Fig. S4.** The effects of TRPM8 agonists on BDL-induced liver fibrosis in mice. H&E, Sirius Red, Masson’s trichrome, and IHC staining for α-SMA and COL1A1 in liver sections of BDL-treated mice (n = 5 per group). Image J was used to quantify positively stained areas. Scale bars, 100 μm. The results are expressed as mean ± SD.

**Additional file 5:** **Fig. S5.** TRPM8 inhibitor attenuates ductular reaction and regulates the expression of S100A9 and HNF4α. **A**,** B** IHC staining for CK19 in liver sections of mice treated with M8-B or DMSO after CCl_4_ or BDL induction (n = 5 per group). Image J was used to quantify positively stained areas. Scale bars, 100 μm. **C**, **D** Expressions of TRPM8, S100A9, and HNF4α were detected by immunoblotting in the liver of mice treated with M8-B or DMSO after CCl_4_ or BDL induction (n = 3 per group). The results are expressed as mean ± SD. *P < 0.05, **P < 0.01.

**Additional file 6:** **Table S1. **Sequences of the primers used in the study. **Table S2. **Demographics and clinicalcharacteristics of fibrosis/cirrhosis patients with different etiologies.

## Data Availability

RNA-seq data were deposited into Sequence Read Archive (SRA) at The National Center for Biotechnology Information (NCBI) with the accession number PRJNA666513. All other relevant data of the study, including clinical data, are available from the corresponding authors upon reasonable request.
