# Supplementary figures and images for: TRPM8 deficiency attenuates liver fibrosis through S100A9-HNF4α signaling
Source: Cell Biosci. 2022 May 7;12:58. doi: 10.1186/s13578-022-00789-4 (PMC9080211; doi:10.1186/s13578-022-00789-4)

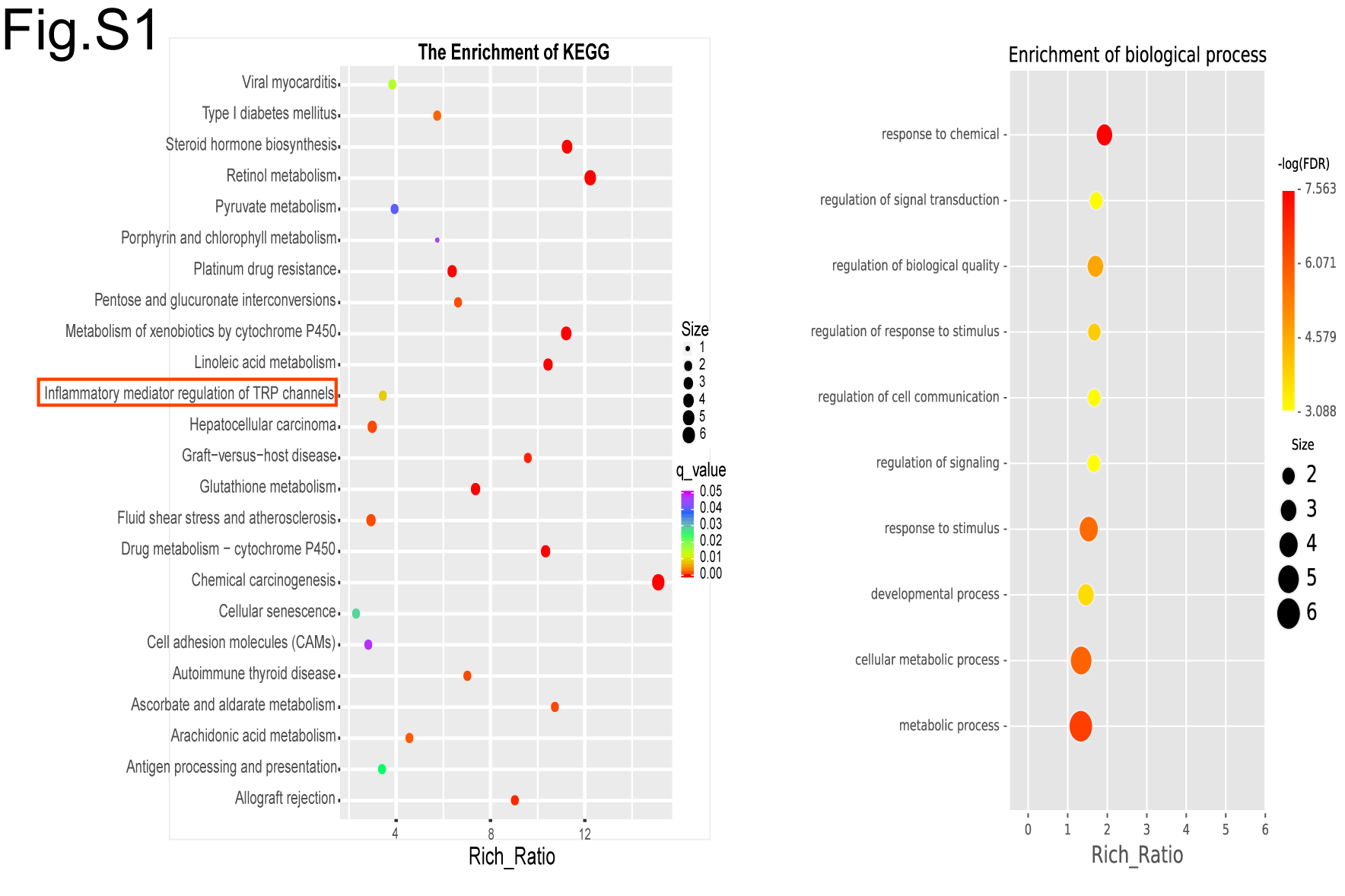

Supplement: Supplementary file 1 — Additional file 1: Fig. S1. KEGG pathway and GO enrichment analysis. [file 13578_2022_789_MOESM1_ESM.tif]

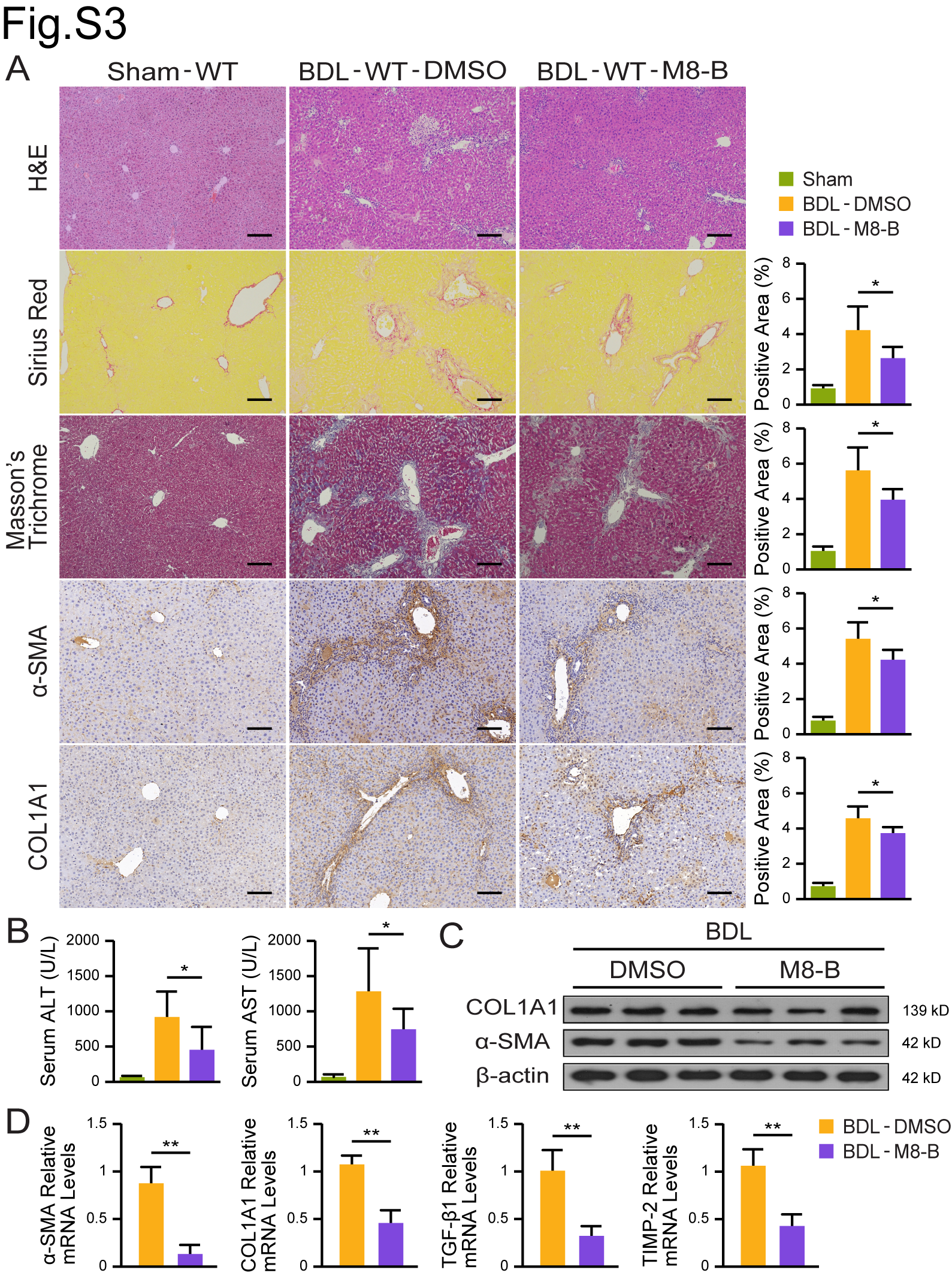

Supplement: Supplementary file 2 — Additional file 2: Fig. S2. The effects of TRPM8 agonists on CCl4-induced liver fibrosis in mice. H&E, Sirius Red, Masson’s trichrome, and IHC staining for α-SMA and COL1A1 in liver sections of CCl4-treated mice (n = 5 per group). Image J was used to quantify positively stained areas. Scale bars, 100 μm. Results are expressed as mean ± SD. [file 13578_2022_789_MOESM2_ESM.tif]

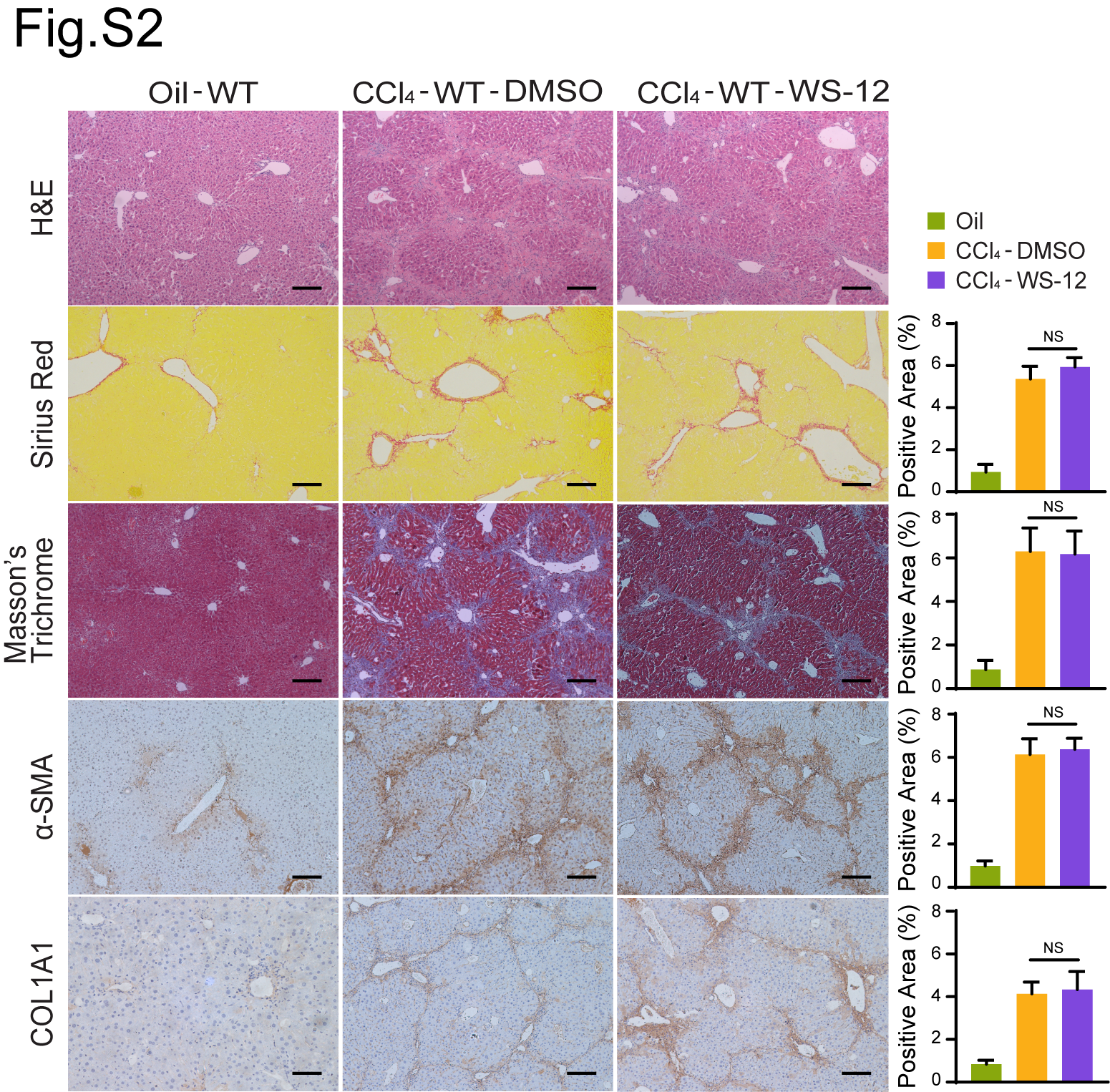

Supplement: Supplementary file 3 — Additional file 3: Fig. S3. TRPM8 inhibitor reduces fibrogenesis in BDL-treated mice. A H&E, Sirius Red, Masson’s trichrome, and IHC staining for α-SMA and COL1A1 in liver sections of BDL-treated mice (n = 5 per group). Image J was used to quantify positively stained areas. Scale bars, 100 μm. B Serum levels of ALT and AST were measured in mice (n = 5 per group). C Expressions of α-SMA and COL1A1 were detected by immunoblotting (n = 3 per group). D Hepatic mRNAs of fibrogenic genes were measured by qRT-PCR assays in mice treated with M8-B or DMSO after BDL induction (n = 5 per group). The results are expressed as mean ± SD. *P < 0.05, **P < 0.01. [file 13578_2022_789_MOESM3_ESM.tif]

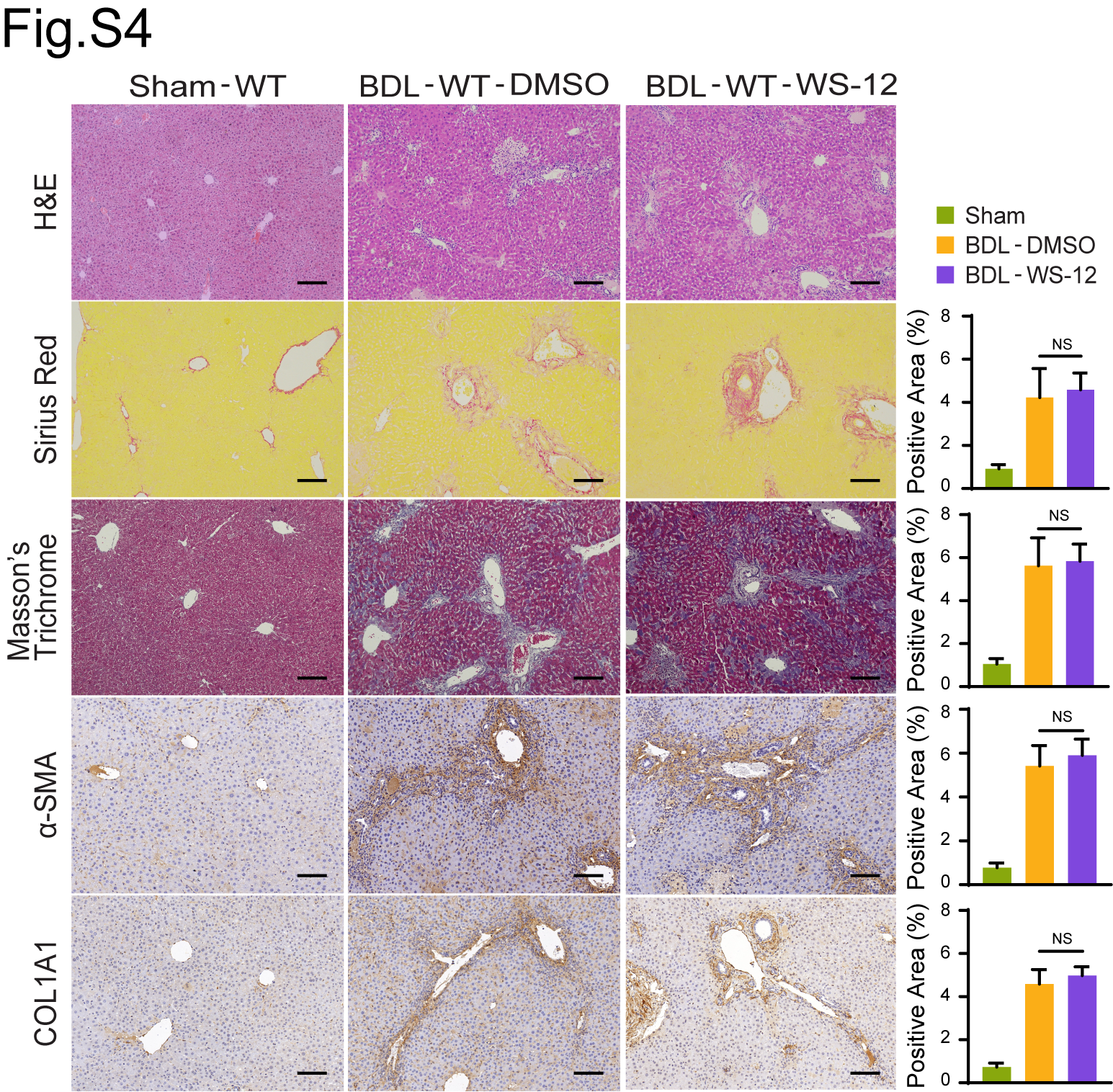

Supplement: Supplementary file 4 — Additional file 4: Fig. S4. The effects of TRPM8 agonists on BDL-induced liver fibrosis in mice. H&E, Sirius Red, Masson’s trichrome, and IHC staining for α-SMA and COL1A1 in liver sections of BDL-treated mice (n = 5 per group). Image J was used to quantify positively stained areas. Scale bars, 100 μm. The results are expressed as mean ± SD. [file 13578_2022_789_MOESM4_ESM.tif]

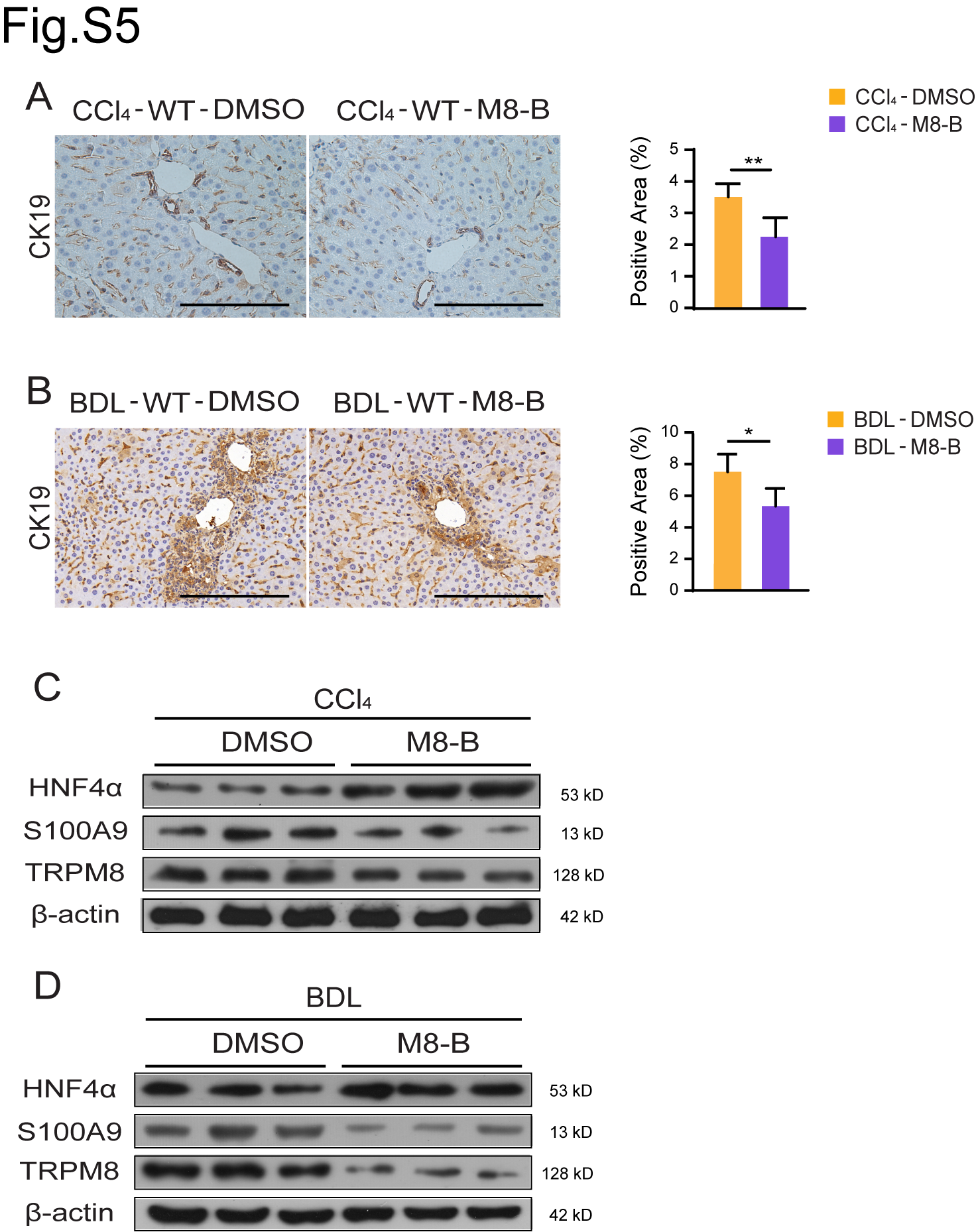

Supplement: Supplementary file 5 — Additional file 5: Fig. S5. TRPM8 inhibitor attenuates ductular reaction and regulates the expression of S100A9 and HNF4α. A, B IHC staining for CK19 in liver sections of mice treated with M8-B or DMSO after CCl4 or BDL induction (n = 5 per group). Image J was used to quantify positively stained areas. Scale bars, 100 μm. C, D Expressions of TRPM8, S100A9, and HNF4α were detected by immunoblotting in the liver of mice treated with M8-B or DMSO after CCl4 or BDL induction (n = 3 per group). The results are expressed as mean ± SD. *P < 0.05, **P < 0.01. [file 13578_2022_789_MOESM5_ESM.tif]
