# Supplementary material for: TRPM8 deficiency attenuates liver fibrosis through S100A9-HNF4α signaling
Source: Cell Biosci. 2022 May 7;12:58. doi: 10.1186/s13578-022-00789-4 (PMC9080211; doi:10.1186/s13578-022-00789-4)
Supplement: Supplementary file 6 — Additional file 6: Table S1. Sequences of the primers used in the study. Table S2. Demographics and clinicalcharacteristics of fibrosis/cirrhosis patients with different etiologies. [file 13578_2022_789_MOESM6_ESM.docx]

**Additional file 6**

**Table S1.** Sequences of the primers used in the study.

| Gene | Primer sequence |
| --- | --- |
| *α-SMA* | F:5′- GCCCCTGAAGAGCATCCGAC-3′  R:5′- CCAGAGTCCAGCACAATACCAGT-3′ |
| *COL1A1* | F:5′- CTGGTGCTCGCGGTAACGAT-3′  R:5′- CAGCACCAGGGTTTCCAGCA-3′ |
| *TGF-β1* | F:5′- CTCCCGTGGCTTCTAGTGC-3′  R:5′- GCCTTAGTTTGGACAGGATCTG-3′ |
| *TIMP-2* | F:5′- TTTTCCAGCCTCTCCCGTCT-3′  R:5′- GTTTGCTCGCTCGGTTTCCT-3′ |
| *IL-6* | F:5′- GACTTCCATCCAGTTGCCTT-3′ |
|  | R:5′- ATGTGTAATTAAGCCTCCGACT-3′ |
| *IL-1β* | F:5′- TGAAATGCCACCTTTTGACAGT-3′ |
|  | R:5′- TTCTCCACAGCCACAATGAGT-3′ |
| *TNFα* | F:5′- AGCACAGAAAGCATGATCCG-3′ |
|  | R:5′- CACCCCGAAGTTCAGTAGACA-3′ |
| *MCP-1* | F:5′- AGCAGCAGGTGTCCCAAA-3′ |
|  | R:5′- CTGAAGACCTTAGGGCAGAT-3′ |
| *BSEP* | F:5′- ATGAAGCCATTGCCGACCAG-3′ |
|  | R:5′- AGGACTTCATCGGCAATAGACCC-3′ |
| *MDR3* | F:5′- AGATCCTCACCAAGCGACT-3′ |
|  | R:5′- ATCACAGCAAGCCTAGACCC-3′ |
| *β-actin* | F:5′- ACATCCGTAAAGACCTCTATGCC-3′ |
|  | R:5′- TTGCCAATGGTGATGACCTG-3′ |

**Table S2.** Demographics and clinical characteristics of fibrosis/cirrhosis patients with different etiologies.

| **Etiology** | **Gender** | **Age (years)** | **ALT (U/L)** | **AST (U/L)** | **ALB (g/L)** | **Tbil (μmol/L)** | **PT (s)** |
| --- | --- | --- | --- | --- | --- | --- | --- |
| Normal 1# | Male | 44 | 12 | 14.1 | 45.7 | 11.8 | 11.5 |
| Normal 2# | Female | 48 | 14.9 | 16.3 | 40.1 | 13.2 | 12.8 |
| Normal 3# | Female | 50 | 17 | 21.3 | 45.1 | 8.7 | 12.7 |
| Normal 4# | Female | 50 | 25.7 | 24.5 | 45.2 | 13.6 | 12.9 |
| Normal 5# | Male | 38 | 18.8 | 19.1 | 39.9 | 18.9 | 13.7 |
| Normal 6# | Female | 37 | 19.9 | 20.1 | 44.6 | 12.6 | 13.3 |
| Normal 7# | Male | 50 | 10.5 | 17.7 | 40.7 | 13.8 | 12.9 |
| Normal 8# | Male | 47 | 23.7 | 22.4 | 48.7 | 9.5 | 12.6 |
| Normal 9# | Female | 36 | 9.8 | 17.4 | 45 | 14.9 | 12.3 |
| Normal 10# | Male | 60 | 21.9 | 17.8 | 40.5 | 21.1 | 13.2 |
| CHB 1# | Male | 42 | 21.7 | 31.6 | 31 | 37.5 | 16.6 |
| CHB 2# | Male | 33 | 152.2 | 59.2 | 37.5 | 469.2 | 22 |
| CHB 3# | Male | 51 | 58 | 163 | 33.6 | 584.4 | 61.6 |
| CHB 4# | Female | 66 | 18.1 | 34 | 32.9 | 61.4 | 15.8 |
| CHB 5# | Male | 50 | 24.6 | 64.1 | 31.1 | 174.5 | 25.1 |
| CHB 6# | Male | 59 | 17.2 | 44.2 | 28.3 | 51.2 | 17.7 |
| CHB 7# | Male | 39 | 80.5 | 119.9 | 27 | 582.9 | 24.5 |
| CHB 8# | Male | 29 | 703.2 | 403 | 33.2 | 189.1 | 29 |
| CHB 9# | Male | 55 | 12.7 | 33.5 | 27.3 | 186.5 | 36.6 |
| CHB 10# | Female | 44 | 34.1 | 87.5 | 30.1 | 438.4 | 30.6 |
| CHB 11# | Male | 61 | 781.1 | 423.2 | 23.3 | 412.5 | 20 |
| CHB 12# | Female | 50 | 34.1 | 67 | 26.4 | 23.7 | 18 |
| CHB 13# | Male | 56 | 1084.1 | 1507.8 | 29.8 | 223.6 | 21.8 |
| CHB 14# | Female | 51 | 15.6 | 39.5 | 34.8 | 62.6 | 19 |
| CHB 15# | Male | 44 | 20.4 | 72 | 24.8 | 176.5 | 22.1 |
| CHB 16# | Male | 54 | 115.5 | 153.7 | 35.7 | 473.4 | 36.6 |
| CHB 17# | Male | 30 | 36.6 | 42.3 | 38.6 | 34.4 | 15.1 |
| CHB 18# | Male | 51 | 27.4 | 56.4 | 27.5 | 63.6 | 17.2 |
| CHB 19# | Male | 41 | 28.3 | 34 | 36.6 | 21.8 | 14.4 |
| CHB 20# | Male | 56 | 150.1 | 175.8 | 35.4 | 658.2 | 25.8 |
| CHB 21# | Male | 50 | 31.5 | 54.9 | 27.5 | 33.9 | 22.3 |
| CHB 22# | Male | 47 | 14.3 | 31.9 | 29.1 | 38.9 | 16.2 |
| CHB 23# | Male | 46 | 52.7 | 85.8 | 39.3 | 332.6 | 24.7 |
| CHB 24# | Male | 58 | 689.3 | 492 | 31.4 | 499.4 | 24.7 |
| CHB 25# | Male | 50 | 462.1 | 120.7 | 26.4 | 271.9 | 29.1 |
| CHB 26# | Male | 46 | 93.6 | 93.5 | 33.2 | 468.9 | 21.7 |
| CHB 27# | Male | 52 | 624.5 | 349.7 | 30.6 | 226.6 | 22 |
| CHB 28# | Male | 32 | 20.3 | 23.5 | 35.3 | 93 | 22.4 |
| CHB 29# | Female | 63 | 95.6 | 219.6 | 20.1 | 345.4 | 16.6 |
| CHB 30# | Male | 51 | 588.8 | 1283 | 24.1 | 326.9 | 24.4 |
| ALD 1# | Male | 49 | 73.6 | 177.8 | 24.2 | 456.5 | 18.1 |
| ALD 2# | Male | 51 | 37.5 | 39.3 | 33.1 | 501.3 | 31.1 |
| ALD 3# | Male | 46 | 24.5 | 41.2 | 32.9 | 221 | 35.6 |
| ALD 4# | Male | 47 | 66.9 | 113 | 29.1 | 48.5 | 17.8 |
| ALD 5# | Male | 63 | 51.6 | 76.4 | 33.5 | 20 | 16.7 |
| ALD 6# | Male | 58 | 30.1 | 47.9 | 29.3 | 77.8 | 15.3 |
| AIH 1# | Female | 21 | 118.1 | 297.3 | 35.9 | 43 | 10.3 |
| AIH 2# | Male | 31 | 76.9 | 237.2 | 29.2 | 158.4 | 19.2 |
| AIH 3# | Male | 59 | 67.7 | 160.7 | 37.8 | 843.9 | 21.2 |
| AIH 4# | Female | 56 | 39.9 | 91.4 | 19.2 | 32.7 | 18 |
